# Isobaric Tags for Relative and Absolute Quantitation in Proteomic Analysis of Potential Biomarkers in Invasive Cancer, Ductal Carcinoma *In Situ*, and Mammary Fibroadenoma

**DOI:** 10.3389/fonc.2020.574552

**Published:** 2020-10-21

**Authors:** Hao Wu, Xian-Yu Zhang, Ming Niu, Fei-Feng Li, Song Gao, Wei Wei, Si-Wei Li, Xing-Da Zhang, Shu-Lin Liu, Da Pang

**Affiliations:** ^1^Genomics Research Center, College of Pharmacy, State-Province Laboratory of Biomedicine and Pharmaceutics of China, Harbin Medical University, Harbin, China; ^2^Sino-Russian Medical Research Center, Harbin Medical University Cancer Hospital, Harbin, China; ^3^Heilongjiang Academy of Medical Sciences, Harbin Medical University, Harbin, China; ^4^HMU-UCCSM Centre for Infection and Genomics, Harbin Medical University, Harbin, China; ^5^Translational Medicine Research and Cooperation Center of Northern China, Heilongjiang Academy of Medical Sciences, Harbin, China; ^6^Department of Breast Surgery, Harbin Medical University Cancer Hospital, Harbin, China; ^7^Department of Microbiology, Immunology and Infectious Diseases, University of Calgary, Calgary, AB, Canada

**Keywords:** invasive breast cancer, breast ductal carcinomas *in situ*, fibroadenomas, isobaric tags for relative and absolute quantitation, proteomics

## Abstract

**Objectives:**

Breast malignancy is a serious threat to women’s health around the world. Following the rapid progress in the field of cancer diagnostics and identification of pathological markers, breast tumor treatment methods have been greatly improved. However, for invasive, ductal carcinomas and mammary fibroadenoma, there is an urgent demand for better breast tumor-linked biomarkers. The current study was designed to identify diagnostic and/or therapeutic protein biomarkers for breast tumors.

**Methods:**

A total of 140 individuals were included, comprising 35 healthy women, 35 invasive breast cancers (IBC), 35 breast ductal carcinomas *in situ* (DCIS), and 35 breast fibroadenoma patients. *Isobaric tags for relative and absolute quantitation (*iTRAQ) proteomic analysis was employed to characterize differentially expressed proteins for potential biomarkers in IBC, DCIS, and fibroadenomas by comparisons with their matched adjacent tissues and/or normal breast tissues. The public databases Metascape and String were used for bioinformatic analyses.

**Results:**

Using the proteomics approach, we identified differentially expressed proteins in tissues of different breast tumors compared to normal/adjacent breast tissues, including 100 in IBC, 52 in DCIS, and 44 in fibroadenoma. Among the 100 IBC differentially expressed proteins, 37 were found to be specific to this type of cancer only. Additionally, four proteins were specifically expressed in DCIS and four in fibroadenoma. Compared to corresponding adjacent tissues and normal breast tissues, 18 step-changing proteins were differentially expressed in IBC, 14 in DCIS, and 13 in fibroadenoma, respectively. Compared to DCIS and normal breast tissues, 65 proteins were differentially expressed in IBC with growing levels of malignancy.

**Conclusions:**

The identified potential protein biomarkers may be used as diagnostic and/or therapeutic targets in breast tumors.

## Introduction

Breast cancer is the most common female malignancy and one of the primary causes of the cancer-associated morbidity and mortality (1). The Global Cancer Statistics estimated 268,600 new breast cancer cases and 41,760 deaths in the United States in 2019 (1). Fibroadenoma is a benign fibroepithelial tumor and is one of the most common breast masses (2), often detected in young females (3, 4). At present, breast tumor diagnostics rely mainly on pathological techniques (5, 6). According to the histopathological findings, breast cancer treatment usually proceeds toward surgical management, followed in most cases by chemotherapies (7). However, the choice of chemotherapies is often complicated by the heterogeneity of the cancers (8).

Application of molecular pathology and whole genome sequencing (WGS) technologies has extended understanding of the biological characteristics of malignant tumors in basic and clinical research (9–11). As a result, the improvements in early cancer diagnostics have resulted in a gradual decrease of breast cancer mortality (12). Simultaneously, great progress has been made in the development of individualized breast cancer treatment (13–15), owing largely to the application of the sequencing technologies. However, the differentially expressed breast tumor genes are generally identified at the DNA or RNA levels, even though protein molecules are responsible for maintenance and functioning of cells, tissues, and the whole organism (16). Therefore, cancer protein biomarkers should be more relevant functional targets.

However, it remains unclear which protein biomarkers may reflect the functional differences between invasive breast cancers (IBC), breast ductal carcinomas *in situ* (DCIS), fibroadenoma tissues, and their adjacent or normal breast tissues. Therefore, to improve diagnostics, it is important to determine which proteins are specifically expressed in IBC, DCIS, or fibroadenoma.

*Isobaric tags for relative and absolute quantitation (*iTRAQ) is a proteomic technique that provides high proteome coverage and labeling efficiency, with no aberrant effects on biochemical properties of the labeled proteins or peptides (17). With the development of iTRAQ reagents, the label-dependent quantitation has been continually improved. This technique is based on the chemical labeling of N-terminus (Nt) and Lys side chains of peptides with unique isobaric tags in up to eight different samples (18). iTRAQ is a promising technique for quantitative analysis of samples. As the protein is directly quantified, it can be compared directly among groups (19).

Many important breast tumor-related proteins have been identified using iTRAQ-based quantitative proteomic analyses (20–22), but few of differential values are known. The aim of the current study is to explore proteins that are differentially expressed in IBC, DCIS, and fibroadenoma. We compared functional proteome between cancerous and matched (adjacent) para-cancerous tissues for the identification of potential molecular targets for breast cancer diagnostics and targeted therapy. In the iTRAQ analysis of proteins isolated from IBC, DCIS, fibroadenoma, corresponding adjacent tissues and normal tissues, we identified several differentially expressed proteins in tissues of different breast tumors, including 100 in IBC, 52 in DCIS, and 44 in fibroadenoma. Our results indicate that these proteins may potentially be used as diagnostic and/or therapeutic targets in breast tumors.

## Materials and Methods

### Collection of Clinical Samples

A total of 140 individuals were included in this study, comprising 35 healthy women, 35 IBC, 35 DCIS, and 35 fibroadenoma patients. All patients were recruited at the Department of Breast Surgery, Harbin Medical University Cancer Hospital, from September to December 2018. The mean age of the individuals was 52.5 years (range, 32–73 years). The mean age at menarche was 15 years (range, 13–17 years). The mean age at first birth was 24.5 years (range, 16–31 years). The mean months of breastfeeding duration was 12 months (range, 3–29 months). Normal breast tissues were obtained using ultrasound-guided, hollow needle puncture technique. IBC, DCIS, fibroadenoma tissues, and their matched adjacent tissues were collected during surgical procedures and were assessed according to the patients’ surgical and pathological analyses. The healthy and fibroadenoma groups had no history of cancers. The breast tumor patients did not receive radiotherapy or chemotherapy treatments prior to surgical operations. The current study was approved by the human ethics committee of Harbin Medical University. All participating patients signed consents and were provided with the relevant information regarding the study.

### Tissue Preparation and Protein Extraction

Tissue samples were sectioned and stored in liquid nitrogen. Proteins were extracted in lysis buffer combined with 1% phenylmethylsulfonyl fluoride (PMSF). Following this, protein samples were sonicated (25%W) on ice for 3 min using a pause pattern (10 s sonication, followed by 10 s break). To precipitate proteins, pre-chilled acetone was added to the samples (5 ml acetone: 1 ml samples). The mixture was stored overnight at −20°C. The suspension was centrifuged at 12,000 g for 10 min at 4°C. Then the supernatant was removed. The precipitate was mixed with 2 ml pre-chilled acetone. The mixture was centrifuged twice at 12,000 g for 15 min at 4°C. The pellet was air-dried at room temperature after the supernatant was removed. Following this, the precipitate was dissolved in 0.5 ml 1M TEAB (Sigma-Aldrich, Australia) and centrifuged for 15 min at room temperature. The collected supernatant was transferred to a fresh 1 ml tube and stored at −20°C until further analysis. Samples from patients diagnosed with the same type of disease were mixed. The protein concentration was quantified using the Bradford Protein Assay (TIANGEN, Beijing, China).

### Isobaric Tags for Relative and Absolute Quantitation 8-Plex Labeling

The iTRAQ labeling procedures were conducted according to the manufacturer’s instructions (AB SCIEX, Shanghai, China). iTRAQ 8-plex experiments were performed to analyze tissue extracts. Two iTRAQ labels, 113 and 114, were chosen for the normal breast tissue analysis. Cancerous and matched adjacent tissues were tested using the following labels (in brackets): IBC tissues (115) and corresponding adjacent tissues (116), DCIS tissues (117) and corresponding adjacent tissues (118), fibroadenoma tissues (119), and corresponding adjacent tissues (121). The samples were analyzed using Triple TOF^TM 4600 (AB SCIEX). Proteins (100 μg) in each group were precipitated using fivefold acetone at −20°C for 1 h. Following this, the mixture was centrifuged at 12,000 g for 10 min at 4°C. The collected suspension of proteins was dried using a vacuum centrifuge. The dried protein was resuspended in 50 μl dissolution buffer, reduced by 4 μl reducing reagent for 1 h at 60°C, and alkylated by 2 μl cysteine blocking reagent for 10 min at room temperature. The protein samples were then digested with 50 μl trypsin (50 ng/μl) at 37°C for 12 h. Tryptic peptides were dried by vacuum centrifugation and labeled using the iTRAQ regents for 2 h at room temperature. Afterward, 100 μl distilled water was added to stop the reaction. After labeling, the samples were mixed at equal volumes, pre-classified into 12 components by high pH RP-HPLC, and then redissolved into 30 μl volume per component. Ten microliters input of each sample was extracted and the samples were dried using a vacuum centrifuge and stored till identification analysis.

### Protein Identification

Protein identification and relative iTRAQ quantification were performed using ProteinPilot™ Software 4.5 (AB SCIEX) and Paragon™ algorithm for the peptide recognition. According to the iTRAQ experimental data obtained from ProteinPilot database, PDST (ProteinPilot Descriptive Statistics Template) data analysis software was used to further collate and analyze the iTRAQ data. Data files were submitted to ProteomeXchange *via* the PRIDE database (**Website:** http://www.ebi.ac.uk/pride; Project Name: iTRAQ in proteomic analysis of potential biomarkers in invasive cancer, ductal carcinoma in situ, and mammary fibroadenoma; Project accession: PXD019963). The screening criteria for this study were set as follows: 1) 114:113 (normal tissues)—between 0.8 and 1.2, *P *> 0.05; 2) 115/116/117/118/119/121:113—two times or above differences were considered as an evident indicator of differentially expressed proteins, *P < *0.05; 3) 115:116; 117:118; 119:121—two times or above differences were considered as an evident indicator of differentially expressed proteins. For other forms of comparison (step-changing proteins or proteins with growing level of malignancy), protein expression data were directly compared.

### Bioinformatics Analysis

The basic properties of differentially expressed proteins were analyzed using Gene Ontology (GO) and protein-protein interaction network at Metascape (https://metascape.org/gp/index.html) and STRING (https://string-db.org/), respectively (23, 24).

## Results

### Differentially Expressed Proteins in Invasive Breast Cancers, Invasive Breast Cancers-Adjacent, and Normal Breast Tissues

We analyzed differentially expressed proteins with ≥2-fold (higher or lower) differences in IBC, IBC-adjacent, and normal breast tissue comparisons. We identified IBC-linked 20 up-regulated proteins (including HSPA4, HSPA9, RRBP1, PGK1, PRKDC, MMP11, and others) compared to both adjacent and normal tissues (**Supplementary Table 1**). In the meantime, analysis of the differences between IBC and both adjacent and normal tissues showed 80 down-regulated proteins (including AZGP1, ALDH1A1, KRT families, APOA families, and others) in IBC tissues (**Supplementary Table 2**).

In the analysis of step-changing proteins, we identified seven proteins (including FLNA, PLEC, MMP11, and others) that were increased in IBC tissues compared to the IBC-adjacent and normal tissues (**Supplementary Table 3**). Additionally, 11 step-changing proteins (including KRT families, PRDX2, CALD1, and others) were decreased in the IBC tissues compared with both cancer-adjacent and normal (**Supplementary Table 3**).

**Figure 1:**
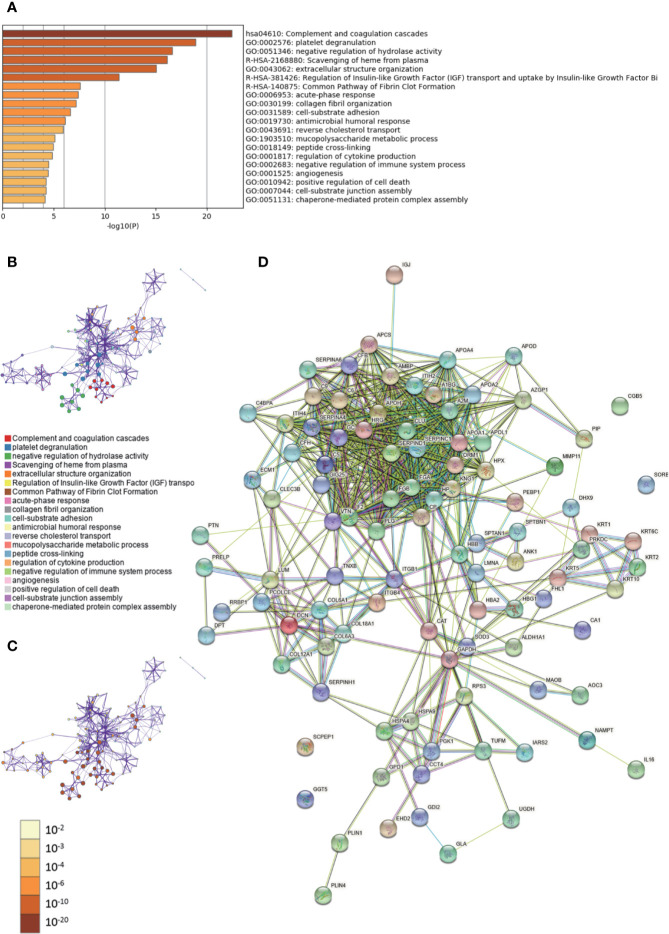
Gene Ontology (GO) and protein–protein interaction (PPI) analyses of differentially expressed proteins in invasive breast cancers (IBC) *vs.* cancer-adjacent and normal breast tissues. **(A)** The GO analysis of 100 differentially expressed proteins using Metascape database. The *x* axis shows the significance which is the value of –log10(P). **(B)** The enrichment network of representative terms is performed with Cytoscape (v3.1.2). Each term is represented by a circle node, the size of which is proportional to the number of input genes falling into that term, and the color represents its cluster identity. Terms with a similarity score > 0.3 are linked by an edge. One term from each cluster is selected to have its term description shown as label. **(C)** The same enrichment network has its nodes colored by p-value. The darker the color, the more statistically significant the node is. **(D)** The PPI network of the differentially expressed proteins was constructed using STRING.

Metascape was used to conduct GO analysis of 100 differentially expressed proteins (**Supplementary Tables 1**, **2**). Moreover, pathway and process enrichment analyses were performed, with the network of the enriched terms. The enriched clusters for differentially expressed proteins in IBC *vs.* IBC-adjacent and normal breast tissues included those of “complement and coagulation cascades,” “regulation of IGF transport,” “negative regulation of immune system process,” “angiogenesis,” and others (**Figures 1A–C**, **Supplementary Data Sheet 1**). In addition, we performed a protein–protein interaction (PPI) enrichment analysis. The PPI network is shown in **Supplementary Figure 1A**. Then, MCODE was used to identify densely connected network components (**Supplementary Figure 1B**). Pathway and process enrichment analysis was independently applied to each MCODE component. The results showed that biological function was mainly related to “scavenging of heme from plasma,” “terminal pathway of complement,” “binding and uptake of ligands by scavenger receptors,” “positive regulation of cytokine production,” “regulation of cytokine production,” “PID UPA UPAR pathway,” “heterotypic cell-cell adhesion,” and “PID intergrin1 pathway” (**Supplementary Figure 1C**). Finally, string database was employed to analyze the interactive network of these proteins. The PPI was mainly concentrated in the relevance among SERPIN families, APO families, FGA, FGB, HRG, GC, CLU, etc. (**Figure 1D**).

### Differentially Expressed Proteins in Ductal Carcinomas *In Situ*, Ductal Carcinomas *In Situ*-Adjacent, and Normal Breast Tissues

For DCIS tissues, compared with both DCIS cancer-adjacent and normal tissues, we detected four up-regulated (GAPDH, HSPA9, CCT4, and SCPEP1) (**Supplementary Table 4**) and 48 down-regulated proteins (including KRT10, APOA1, ALDH1A1, and others) (**Supplementary Table 5**) in DCIS tissues.

Step-changing proteins were identified in DCIS compared with cancer-adjacent and normal tissues, including increased expression of STAM, ARF5, ANXA6, SCPEP1, ME2, and WFS1 (**Supplementary Table 6**), and decreased expression of KRT1, KRT10, KRT6E, APOA1, DSP, LUM, ANK1, and F2 in DCIS tissues (**Supplementary Table 6**).

**Figure 2:**
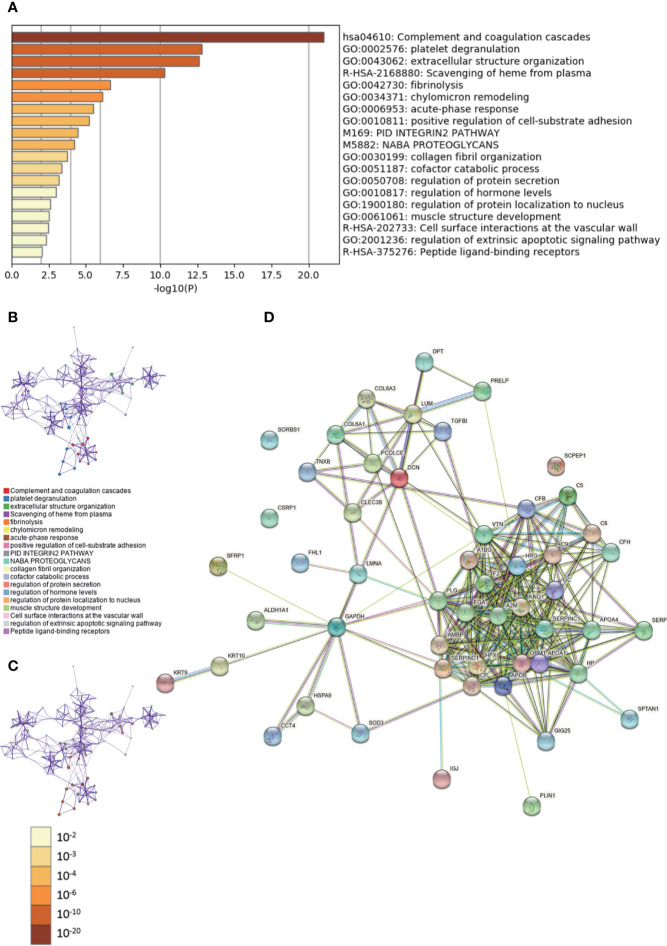
Gene Ontology (GO) and protein–protein interaction (PPI) analyses of differentially expressed proteins in DCIS *vs.* ductal carcinomas *in situ* (DCIS)-adjacent and normal breast tissues. **(A)** The GO analysis of 52 differentially expressed proteins using Metascape database. The *x* axis shows the significance which is the value of –log10(P). **(B)** The enrichment network of representative terms is performed with Cytoscape (v3.1.2). Each term is represented by a circle node, the size of which is proportional to the number of input genes falling into that term, and the color represents its cluster identity. Terms with a similarity score > 0.3 are linked by an edge. One term from each cluster is selected to have its term description shown as label. **(C)** The same enrichment network has its nodes colored by p-value. The darker the color, the more statistically significant the node is. **(D)** The PPI network of the differentially expressed proteins was constructed using STRING.

During GO analysis, we defined the enriched clusters for 52 differentially expressed proteins (**Supplementary Tables 4**, **5**). The enriched clusters for differentially expressed proteins in DCIS *vs.* adjacent and normal breast tissues included those of “complement and coagulation cascades,” “extracellular structure organization,” “positive regulation of cell-substrate adhesion,” “regulation of protein secretion,” “regulation of hormone levels,” and others (**Figures 2A–C**, **Supplementary Data Sheet 2**). Then, we performed a Metascape PPI enrichment analysis. The PPI network and MCODE components identiﬁed in the lists are shown in **Supplementary Figures 2A, B**. The biological function was mainly related to “regulation of complement cascade,” “complement cascade” and “terminal pathway of complement” (**Supplementary Figure 2C**). Additionally, the String database was used to analyze the interaction network of these proteins. The PPI was mainly concentrated in the relevance among SERPIN families, APO families, FGA, A2M, KNG1, HRG, GC, etc. (**Figure 2D**).

### Differentially Expressed Proteins in Fibroadenoma, Fibroadenoma-Adjacent, and Normal Breast Tissues

For fibroadenoma tissues, compared with both fibroadenoma-adjacent and normal tissues, there were six up-regulated (ANXA6, VCP, SERPINH1, galactosidase alpha, NNT, and MMP11) (**Supplementary Table 7**) and 38 down-regulated (KRT10, KRT1, ALDH1A1, ECM1, and others) (**Supplementary Table 8**) proteins in fibroadenoma.

The step-changing proteins were detected in fibroadenoma, fibroadenoma-adjacent and normal breast tissues. We detected an increase in the expression of six proteins (NUDT19, FBN1, NONO, FLNA, SCPEP1, and galactosidase alpha) in fibroadenoma (**Supplementary Table 9**). Furthermore, the expression of seven step-changing proteins (KRT10, KRT1, KRT6E, PLIN1, HBA2, FHL1, and ME2) were decreased in fibroadenoma tissues compared to fibroadenoma adjacent and normal tissues (**Supplementary Table 9**).

**Figure 3:**
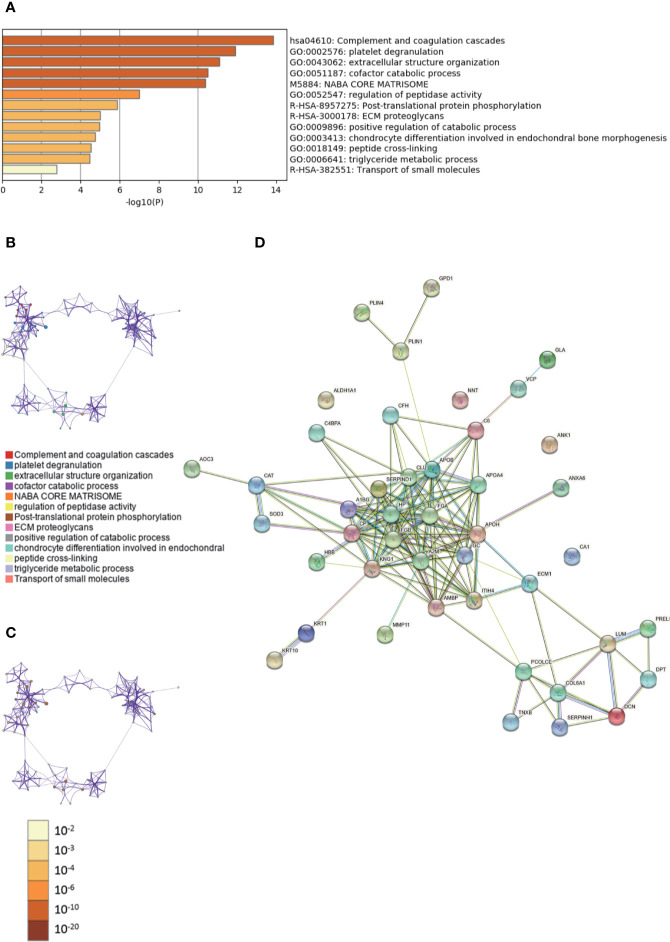
Gene Ontology (GO) and protein–protein interaction (PPI) analyses of differentially expressed proteins in fibroadenoma *vs.* fibroadenoma-adjacent and normal breast tissues. **(A)** The GO analysis of 44 differentially expressed proteins using Metascape database. The *x* axis shows the significance which is the value of –log10(P). **(B)** The enrichment network of representative terms is performed with Cytoscape (v3.1.2). Each term is represented by a circle node, the size of which is proportional to the number of input genes falling into that term, and the color represents its cluster identity. Terms with a similarity score > 0.3 are linked by an edge. One term from each cluster is selected to have its term description shown as label. **(C)** The same enrichment network has its nodes colored by p-value. The darker the color, the more statistically significant the node is. **(D)** The PPI network of the differentially expressed proteins was constructed using STRING.

The enriched clusters for differentially expressed proteins in **Supplementary Tables 7**, **8** were identified using GO analysis. The enriched clusters for differentially expressed proteins in fibroadenoma *vs.* adjacent and normal breast tissues included those of “complement and coagulation cascades,” “extracellular structure organization,” “cofactor catabolic process,” “ECM proteoglycans,” “triglyceride metabolic process,” “regulation of angiogenesis,” and others (**Figures 3A–C**, **Supplementary Data Sheet 3**). The String database was used to analyze the interaction network of these proteins. The PPI was mainly concentrated in the relevance among SERPIND1, APO families, FGA, FGB, A2M, KNG1, HP, GC, etc. (**Figure 3D**).

### Specific Up- or Down-Regulated Differentially Expressed Proteins in Invasive Breast Cancers, Ductal Carcinomas *In Situ*, and Fibroadenoma Tissues

We further determined the proteins that were differentially expressed in IBC, DCIS, and fibroadenoma in a tissue-specific manner. Intersections of up-regulated and down-regulated proteins for IBC, DCIS, and fibroadenoma tissues were determined. We found that 14 proteins (hCG, TUFM, HSPA4, RRBP1, RPS3, PGK1, PRKDC, COL12A1, GDI2, IARS2, DHX9, GLA, UGDH, and NAMPT) were specifically up-regulated in IBC tissues, and 23 proteins (PIP, APOD, KRT2, APOA2, KRT6E, IL16, AZGP1,HBA2, KRT5, HBG1, ITIH2, SPTBN1, COL18A1,SERPINA4, PEBP1, APOL1, GGT5, MAOB, ITGB4,EHD2, APCS, ITGB1, and PTN) were specifically down-regulated in IBC tissues. In DCIS tissues, we found four specifically down-regulated proteins (SFRP1, KRT9, TGFBI, CSRP1) but not any up-regulated proteins. In fibroadenoma, four proteins were specifically up-regulated (ANXA6, VCP, Galactosidase alpha, and NNT), while no down-regulated proteins were not found (**Supplementary Table 10**).

### Differentially Expressed Proteins Associated With Growing Levels of Malignancy in Invasive Breast Cancers and Ductal Carcinomas *In Situ* Tissues

As very few specific proteins from DCIS or IBC have been reported to date, we focused on those that were differentially expressed along with the growing levels in the degree of malignancy of the tumors. According to the data shown in **Supplementary Tables 4**, **5**, we detected proteins that were expressed higher or lower in DCIS tissues compared to that in normal breast tissues. Following this, the identified proteins were compared with proteins in the IBC tissues. We found that increased expression of 10 proteins (RRBP1, HSPA9, PGK1, GAPDH, IARS2, CCT4, Galactosidase alpha, NAMPT, NNT, WFS1) coincided with the progression of malignancy (**Supplementary Table 11**). We also screened proteins whose expression declined gradually. We found that expression levels of 55 proteins (KRT10, KRT1, PIP, and others) were decreased along with the increase in a degree of malignancy (**Supplementary Table 11**). These proteins may play important roles in the development of breast cancer as cancer promoters or suppressors.

**Figure 4:**
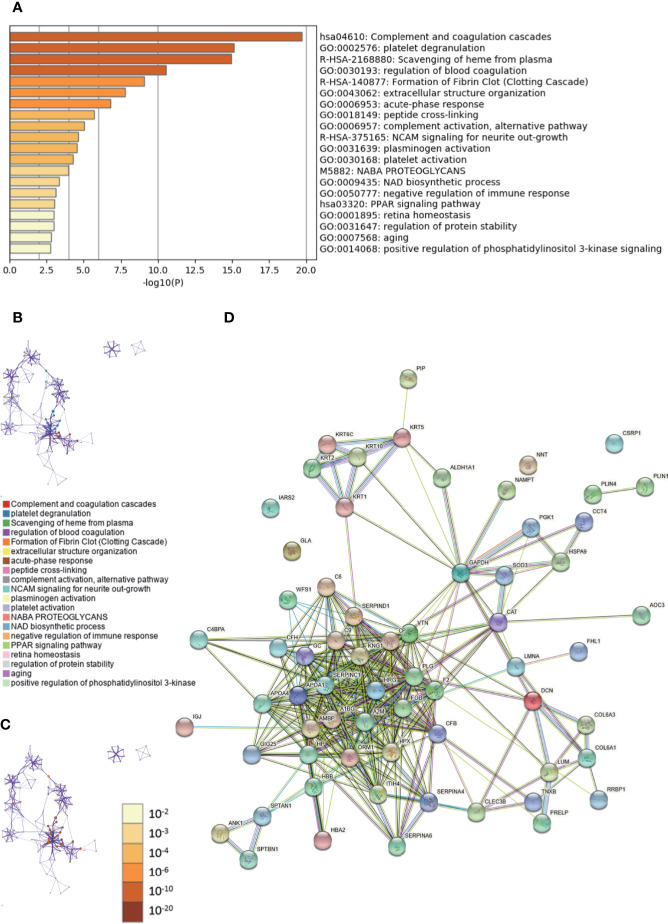
Gene Ontology (GO) and protein–protein interaction (PPI) analyses of differentially expressed proteins associated with growing level of malignancy in invasive breast cancers (IBC) and ductal carcinomas *in situ* (DCIS) tissues. **(A)** The GO analysis of 65 differentially expressed proteins using Metascape database. The *x* axis shows the significance which is the value of –log10(P). **(B)** The enrichment network of representative terms is performed with Cytoscape (v3.1.2). Each term is represented by a circle node, the size of which is proportional to the number of input genes falling into that term, and the color represents its cluster identity. Terms with a similarity score > 0.3 are linked by an edge. One term from each cluster is selected to have its term description shown as label. **(C)** The same enrichment network has its nodes colored by p-value. The darker the color, the more statistically significant the node is. **(D)** The PPI network of the differentially expressed proteins was constructed using STRING.

The enriched clusters for these proteins included those of “complement and coagulation cascades,” “extracellular structure organization,” “PPAR signal pathway,” “regulation of protein stability,” and others (**Figures 4A–C**, **Supplementary Data Sheet 4**). The PPI network and MCODE components identiﬁed in the lists are shown in **Supplementary Figures 3A, B**. The biological function was mainly related to “scavenging of heme from plasma,” “binding and uptake of ligands by scavenger receptors,” “cornification,” “interaction between L1 and ankyrins,” “COPI-mediated anterograde transport,” and “L1CAM interactions” (**Supplementary Figure 3C**). The String database was used to analyze the interaction network of these proteins. The PPI was mainly concentrated in the relevance among SERPIN families, APO families, HRG, A1BG, A2M, FGB, AMBP, etc. (**Figure 4D**).

## Discussion

**Figure 5:**
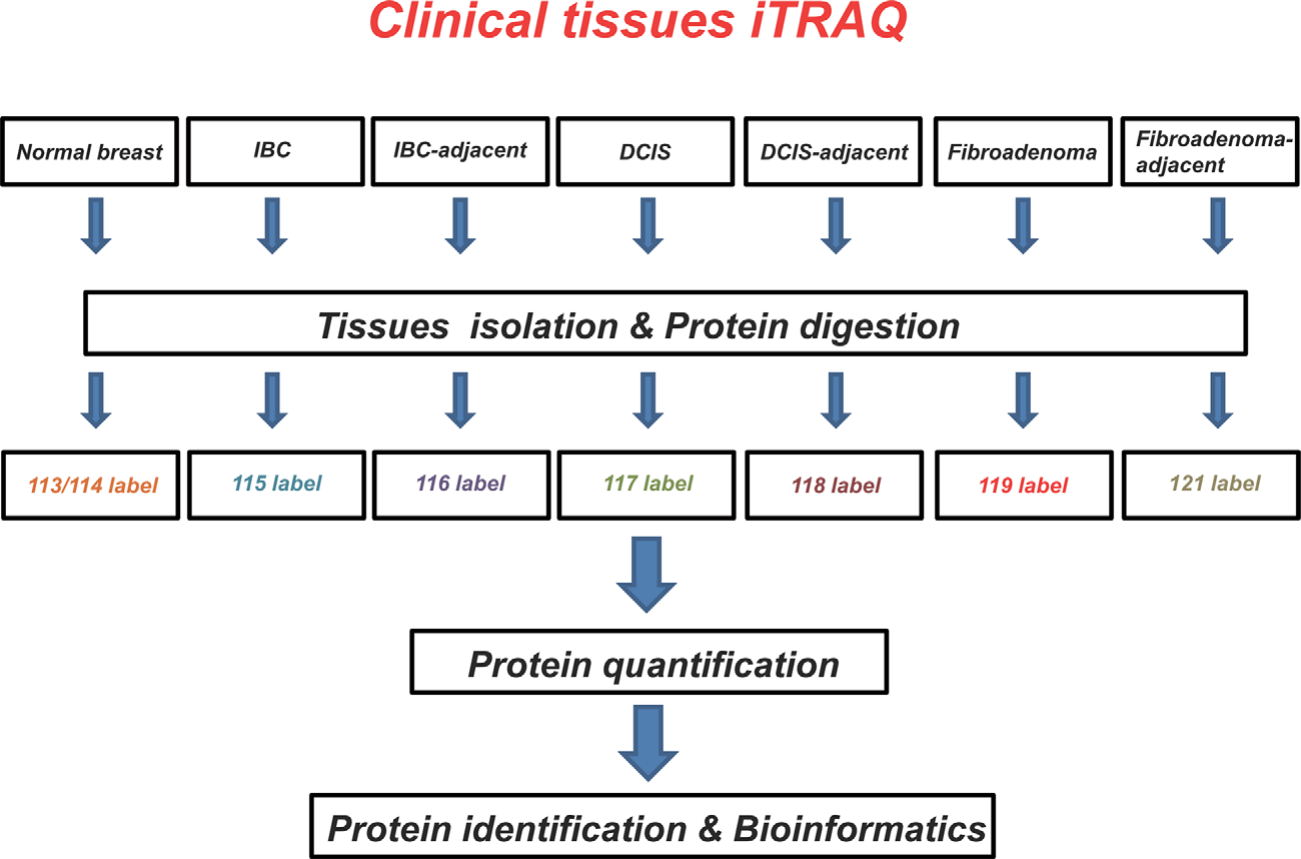
Schematic overview of the strategies used for isobaric tags for relative and absolute quantitation (iTRAQ) analyses. Using iTRAQ approach, we identified potential protein biomarkers that might be used as diagnostic and/or therapeutic targets in breast tumors.

In this study, we identified differentially expressed proteins in breast tumor tissues by the iTRAQ technology. Our work demonstrated that the selected proteins are important for tumor growth survival or spreading. To our best knowledge, this study was the first performed for assessing and comparing protein changes between normal, IBC, DCIS, mammary fibroadenoma, and matched adjacent tissues, providing new potential biomarkers of protein molecules for diagnosis/treatment of breast tumor patients (**Figure 5**).

Among the analyzed IBC tissues, we found 20 proteins (including HSPA4, HSPA9, RRBP1, PGK1, PRKDC, MMP11, and others) that showed more than a two-fold increase in expression relative to adjacent or normal breast tissues. Many of the identified proteins have been previously reported to be involved in oncogenesis and our results are in line with such findings. For instance, the higher expression of HSPA4 in cancers was associated with metastasis and poor prognosis in breast cancer patients (25). HSPA9, a member of the heat shock protein family, regulates Raf/MEK/extracellular signal-regulated kinase (ERK) and is associated with tumorigenesis, especially drug resistance in breast cancer treatment (26, 27). The highly expressed PRKDC could promote breast cancer cell growth and was associated with poor survival of the patients (28). MMP11 has also been reported as a novel prognostic factor in breast cancer (29). Associations with cancer progression have also been previously reported for RRBP1 (30), PGK1 (31), and C1QBP (32, 33) by us, and our results in this study provided further supports, with **t**he expression levels of the above listed proteins highly correlated with breast cancer development and progression. Some of the screened proteins, like RPS3, GDI2, IARS2, were less investigated and their cancer-related mechanism and roles remain unclear in breast cancers. These proteins have a potential to serve as biomarkers and should be explored as diagnostic or therapeutic targets in breast cancers. Further investigation of these proteins may prove their potential use as anti-cancer targets in clinical treatment.

Tissue-specific proteins have been an extensively investigated topic in cancer research. We identified differentially expressed molecules that were specifically up- or down-regulated in IBC, DCIS, and fibroadenoma in the current study. Specifically, expression levels of HSPA4, RRBP1, PGK1, PRKDC, GLA, UGDH, and NAMPT were highly increased in IBC. They have been reported to be involved in the development of breast cancer. Therefore, we suggest that these proteins may be used as diagnostic markers in preoperative puncture-based pathological tests to distinguish IBC from DCIS. The set of proteins may provide a rich and robust reference for preoperative diagnosis and help to formulate future surgical plans.

The transition from DCIS to IBC should be clearly defined to direct clinical decisions during breast cancer development. Comparison of differential expression levels of proteins between IBC, DCIS, and normal tissues is therefore of great importance. A set of 10 up-regulated (RRBP1, PGK1, NAMPT, and others) and 55 down-regulated (KRT families, SERPIN families, ALDH1A1, and others) proteins with gradient changes identified in this study may prove to be useful biomarkers and further research may elucidate whether continuous changes in expression of these proteins may play significant roles in the progression of the breast malignancy.

Several proteins identified here to be specific are worth further investigating. Secreted proteins such as MMP11 (34) and COL12A1 (34) were shown to play important roles in breast carcinogenesis and identified as highly expressed in our study. Therefore, they can be potential blood markers for the diagnosis of early breast cancer. We also detected the higher expression of HCG in IBC. HCG can be used as a tumor marker as the protein is involved in regulation of tumorigenesis (35). Meanwhile, it has been shown to play an important role in treatment of breast cancer (35). The anti- or pro-tumorigenesis effects of HCG in breast cancer deserve to be further explored. FLNA has been reported as oncogene in some cancers (36) but as tumor suppressor in others (37, 38). Our results showed that it was higher in cancer tissues compared with adjacent tissues. Therefore, the role of FLNA as cancer promoting or suppressing effector requires further clarifications. Additionally, we noticed that the expression level of GAPDH, a reference gene commonly used in research, was gradually increased following the increases in the degree of malignancy. GAPDH was shown to be involved in regulation of tumor cell glycolysis and autophagy (39) and was also linked to microenvironment-associated hypoxia (40). Notably, most solid tumors are exposed to hypoxic conditions (39). Therefore, whether GAPDH can be used rightfully as a reference protein requires further investigations in breast cancers.

WGS technologies have played important roles in exploring the genesis and development of breast cancer (41). However, there is continuous generation and degradation of RNA during its biological functioning. Besides, RNA undergoes multi-step processing from mRNA to protein translation. Therefore, it is difficult to determine the role that RNA plays in the development of tumors, especially during the change of tumor properties. Compared to RNA, proteins are more stable molecules involved in direct execution of specific biological functions. The level of differentially expressed proteins in breast cancer remains unclear at different stages of oncogenesis. The identification of proteins responsible for cancer development is an important step for understanding of oncogenesis. Notably, we mixed the same type of samples together during the processing, which resulted in elimination of individual differences between patients. This technical procedure might be associated with certain defects in the experiment. The number of collected samples per group was rather small.

In summary, protein mass spectrometry helps to identify new protein targets for breast cancer. Regarding the clinical significance of our data, we suggest that the identified proteins be further confirmed as new potential biomarkers for breast tumor. Based on the current clinical application and the development of biomedical sciences, formalin-fixed and paraffin-embedded (FFPE) tissues or blood samples are much more easily collected and should be used for protein detection and identification by iTRAQ (42, 43). Seeing that these samples have high application values with the advantages of clear history, diagnosis and detailed clinical data, proteomics (iTRAQ) analyses on these samples from breast tumor patients are of great significance in the study of disease mechanisms and new biomarkers discovery. The aim of the present study was mainly to identify differentially expressed proteins and to pave the way for further studies on pathogenesis or evolution mechanisms in breast tumor. However, samples from larger groups involving larger number of participants should be collected to verify the detected expression differences. Then a strict training, test, and validation process will be conducted. Besides, it is notable that the current trend in modern cancer medicine is oriented toward individualized therapy. The analysis of each patient’s samples separately will provide more specific molecular targets and improve the treatment outcome.

## Data Availability Statement

The datasets presented in this study can be found in online repositories. The names of the repository/repositories and accession number(s) can be found below: ProtemoeXchange *via* the PRIDE database (http://www.ebi.ac.uk/pride) (PXD019963).

## Ethics Statement

The studies involving human participants were reviewed and approved by The Medical Ethics Committee of Harbin Medical University Cancer Hospital. The patients/participants provided their written informed consent to participate in this study.

## Author Contributions

DP, HW, and X-YZ designed experiments. MN and WW carried out the experiments. S-LL, HE, F-FL, and SG analyzed experimental results. S-LL, HW, S-WL, and X-DZ wrote the manuscript. DP, HW, X-YZ, and MN funded the research. All authors contributed to the article and approved the submitted version.

## Funding

This work was supported by the National Natural Science Foundation of China, DP, Grant Numbers: 81972706; Heilongjiang Health and Family Planning Commission Foundation, HW, Grant Numbers: 2019-056; Haiyan Fund Project of Harbin Medical University Cancer Hospital, HW, Grant Numbers: JJQN 2018-06; Heilongjiang Province Applied Technology Research and Development, X-YZ, Grant Numbers: GA20C016; Haiyan Fund Project of Harbin Medical University Cancer Hospital, X-YZ, Grant Numbers: JJZD 2020-08; Heilongjiang Health and Family Planning Commission Foundation, MN, Grant Numbers: 2016-085; Heilongjiang Postdoctoral Foundation, China, MN, Grant Numbers: LBH-Z16246; Innovation Scientific Research Fund of Harbin Medical University, MN, Grant Numbers: 2017-LCZX81.

## Conflict of Interest

The authors declare that the research was conducted in the absence of any commercial or financial relationships that could be constructed as a potential conflict of interest.

## Supplementary Material

Supplementary Figure 1 PPI analyses of differentially expressed proteins in IBC vs cancer-adjacent and normal breast tissues using Metascape. **(A)** PPI network of proteins encoded by differentially expressed proteins. **(B)** Modules selected from PPI network using MCODE. Nodes represent differentially expressed proteins; lines represent interaction relationships between nodes. **(C)** Independent functional enrichment analysis of MCODE components.

Supplementary Figure 2 PPI analyses of differentially expressed proteins in DCIS vs cancer-adjacent and normal breast tissues using Metascape. **(A)** PPI network of proteins encoded by differentially expressed proteins. **(B)** Modules selected from PPI network using MCODE. Nodes represent differentially expressed proteins; lines represent interaction relationships between nodes. **(C)** Independent functional enrichment analysis of MCODE components.

Supplementary Figure 3 PPI analyses of differentially expressed proteins associated with growing level of malignancy in IBC and DCIS tissues using Metascape. **(A)** PPI network of proteins encoded by differentially expressed proteins. **(B)** Modules selected from PPI network using MCODE. Nodes represent differentially expressed proteins; lines represent interaction relationships between nodes. **(C)** Independent functional enrichment analysis of MCODE components.

Supplementary Table 1 20 up-regulated proteins of IBC tissues compared to adjacent and normal tissues. Differentially expressed proteins with ≥2-fold higher differences in IBC compared to both IBC-adjacent and normal tissues were screened.

Supplementary Table 2 80 Down-regulated proteins of IBC tissues compared to adjacent and normal tissues. Differentially expressed proteins with ≥2-fold lower differences in IBC compared to both IBC-adjacent and normal tissues were screened.

Supplementary Table 3 Step-changing of 7 up-regulated and 11 down-regulated proteins in IBC, adjacent and normal tissues. Differentially expressed proteins with ≥2-fold (higher or lower) differences in IBC or IBC-adjacent tissues compared to normal tissues were screened (*P*<0.05). Next, proteins with higher or lower differences in IBC compared to IBC-adjacent tissues were further screened.

Supplementary Table 4 4 up-regulated proteins of DCIS tissues compared to adjacent and normal tissues. Differentially expressed proteins with ≥2-fold higher differences in DCIS compared to both DCIS-adjacent and normal tissues were screened.

Supplementary Table 5 48 down-regulated proteins of DCIS tissues compared to adjacent and normal tissues. Differentially expressed proteins with ≥2-fold lower differences in DCIS compared to both DCIS-adjacent and normal tissues were screened.

Supplementary Table 6 Step-changing of 6 up-regulated and 8 down-regulated proteins in DCIS, adjacent and normal tissues. Differentially expressed proteins with ≥2-fold (higher or lower) differences in DCIS or DCIS-adjacent tissues compared to normal tissues were screened (*P*<0.05). Next, proteins with higher or lower differences in DCIS compared to DCIS-adjacent tissues were further screened.

Supplementary Table 7 6 up-regulated proteins of fibroadenoma tissues compared to adjacent and normal tissues. Differentially expressed proteins with ≥2-fold higher differences in fibroadenoma compared to both fibroadenoma-adjacent and normal tissues were screened.

Supplementary Table 8 38 down-regulated proteins of fibroadenoma tissues compared to adjacent and normal tissue. Differentially expressed proteins with ≥2-fold lower differences in fibroadenoma compared to both fibroadenoma-adjacent and normal tissues were screened.

Supplementary Table 9 Step-changing of 6 up-regulated and 7 down-regulated proteins in fibroadenoma, adjacent and normal tissues. Differentially expressed proteins with ≥2-fold (higher or lower) differences in fibroadenoma or fibroadenoma-adjacent tissues compared to normal tissues were screened (*P*<0.05). Next, proteins with higher or lower differences in fibroadenoma compared to fibroadenoma-adjacent tissues were further screened.

Supplementary Table 10 Specific up- or down-regulated proteins in IBC, DCIS and fibroadenoma tissues. Differentially expressed proteins with ≥2-fold (higher or lower) differences in IBC/DCIS/fibroadenoma compared to matched tumor-adjacent tissues and normal tissues were screened. Intersections of upregulated and downregulated proteins for IBC, DCIS, and fibroadenoma tissues were determined using Wayne chart way.

Supplementary Table 11 10 up-regulated and 55 down-regulated proteins with growing level of malignancy in IBC and DCIS tissues. Differentially expressed proteins with ≥2-fold (higher or lower) differences in IBC or DCIS tissues compared to normal tissues were screened (*P*<0.05). Next, proteins with higher or lower differences in IBC compared to DCIS tissues were further screened.

Supplementary Data sheet 1 Annotation and enrichment informations of up/down-regulated proteins of IBC tissues compared to adjacent and normal tissues.

Supplementary Data sheet 2 Annotation and enrichment informations of up/down-regulated proteins of DCIS tissues compared to adjacent and normal tissues.

Supplementary Data sheet 3 Annotation and enrichment informations of up/down-regulated proteins of fibroadenoma tissues compared to adjacent and normal tissues.

Supplementary Data sheet 4 Annotation and enrichment informations of IBC vs DCIS-related proteins.

## Abbreviations

IBC, invasive breast cancer; DCIS, breast ductal carcinomas *in situ*; iTRAQ, *isobaric tags for relative and absolute quantitation*; WGS, whole genome sequencing; A1BG, alpha-1B-glycoprotein; A2M, alpha-2-macroglobulin; ALDH1A1, aldehyde dehydrogenase 1 family member A1; APCS, antigen-presenting cells; APOA, apolipoprotein A; APOA1, apolipoprotein A1; ARF5, ADP ribosylation factor 5; AMBP, alpha-1-microglobulin/bikunin precursor; ANK1, ankyrin 1; ANXA6, annexin A6; APOA2, apolipoprotein A2; APOD, apolipoprotein D; APOL1, apolipoprotein L1; AZGP1, alpha-2-glycoprotein 1, zinc-binding; CALD1, caldesmon 1; CCT4, chaperonin containing TCP1 subunit 4; CLU, clusterin; COL12A1, collagen type XII alpha 1 chain; COL18A1, collagen type XVIII alpha 1 chain; DHX9, DExH-box helicase 9; DSP, desmoplakin; ECM1, extracellular matrix protein 1; EHD2, EH domain containing 2; F2, coagulation factor II, thrombin; FBN1, fibrillin 1; FLNA, filamin A; FGA, fibrinogen alpha chain; FGB, fibrinogen beta chain; FHL1, four and a half LIM domains 1; GAPDH, glyceraldehyde-3-phosphate dehydrogenase; GC, GC vitamin D binding protein; GDI2, GDP dissociation inhibitor 2; GGT5, gamma-glutamyltransferase 5; GLA, galactosidase alpha; CSRP1, cysteine and glycine rich protein 1; HBA2, hemoglobin subunit alpha 2; hCG, human chorionic gonadotropin; HP, haptoglobin; HRG, histidine rich glycoprotein; HSPA4, heat shock protein family A member 4; HSPA9, heat shock protein family A member 9; IARS2, isoleucyl-TRNA synthetase 2; IL16, interleukin 16; ITGB1, integrin subunit beta 1; ITGB4, integrin subunit beta 4; ITIH2, inter-alpha-trypsin inhibitor heavy chain 2; HBG1, hemoglobin subunit gamma 1; KNG1, kininogen 1; KRT, keratin; LUM, lumican; MAOB, monoamine oxidase B; ME2, malic enzyme 2; MMP11, matrix metalloproteinase 11; NAMPT, nicotinamide phosphoribosyltransferase; NONO, non-POU domain containing octamer binding; NNT, nicotinamide nucleotide transhydrogenase; NUDT19, nudix hydrolase 19; RRBP1, ribosome binding protein 1; RPS3, ribosomal protein S3; PEBP1, phosphatidylethanolamine binding protein 1; PGK1, phosphoglycerate kinase 1; PIP, prolactin induced protein; PLEC, plectin; PLIN1, perilipin1; PRDX2, peroxiredoxin 2; PRKDC, protein kinase, DNA-activated, catalytic subunit; PTN, pleiotrophin; SCPEP1, serine carboxypeptidase 1; SERPINA4, serpina family A member 4; SERPINH1, serpina family H member 1; SFRP1, secreted frizzled related protein 1; SPTBN1, spectrin beta, non-erythrocytic 1; STAM, signal transducing adaptor molecule; TGFBI, transforming growth factor beta induced; TUFM, Tu translation elongation factor, mitochondrial; UGDH, UDP-glucose 6-dehydrogenase; WFS1, wolframin ER transmembrane glycoprotein; VCP, valosin containing protein.
