# Supplementary material for: Isobaric Tags for Relative and Absolute Quantitation in Proteomic Analysis of Potential Biomarkers in Invasive Cancer, Ductal Carcinoma In Situ, and Mammary Fibroadenoma
Source: Front Oncol. 2020 Oct 21;10:574552. doi: 10.3389/fonc.2020.574552 (PMC7640741; doi:10.3389/fonc.2020.574552)

a

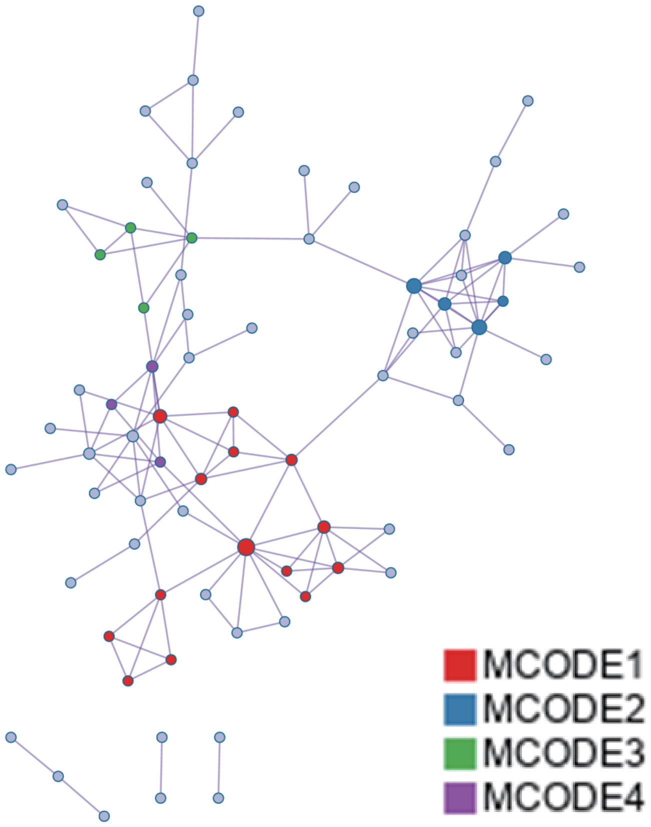

c

Color	MCODE	GO	Description	Log10(P)
	MCODE_1	R-HSA-2168880	Scavenging of heme from plasma	-13.4
	MCODE_1	R-HSA-166665	Terminal pathway of complement	-11.3
	MCODE_1	R-HSA-2173782	Binding and Uptake of Ligands by Scavenger Receptors	-10.6
	MCODE_3	GO:0001819	positive regulation of cytokine production	-4.5
	MCODE_3	GO:0001817	regulation of cytokine production	-3.9
	MCODE_4	M174	PID UPA UPAR PATHWAY	-8.3
	MCODE_4	GO:0034113	heterotypic cell-cell adhesion	-7.8
	MCODE_4	M18	PID INTEGRIN1 PATHWAY	-7.7

b

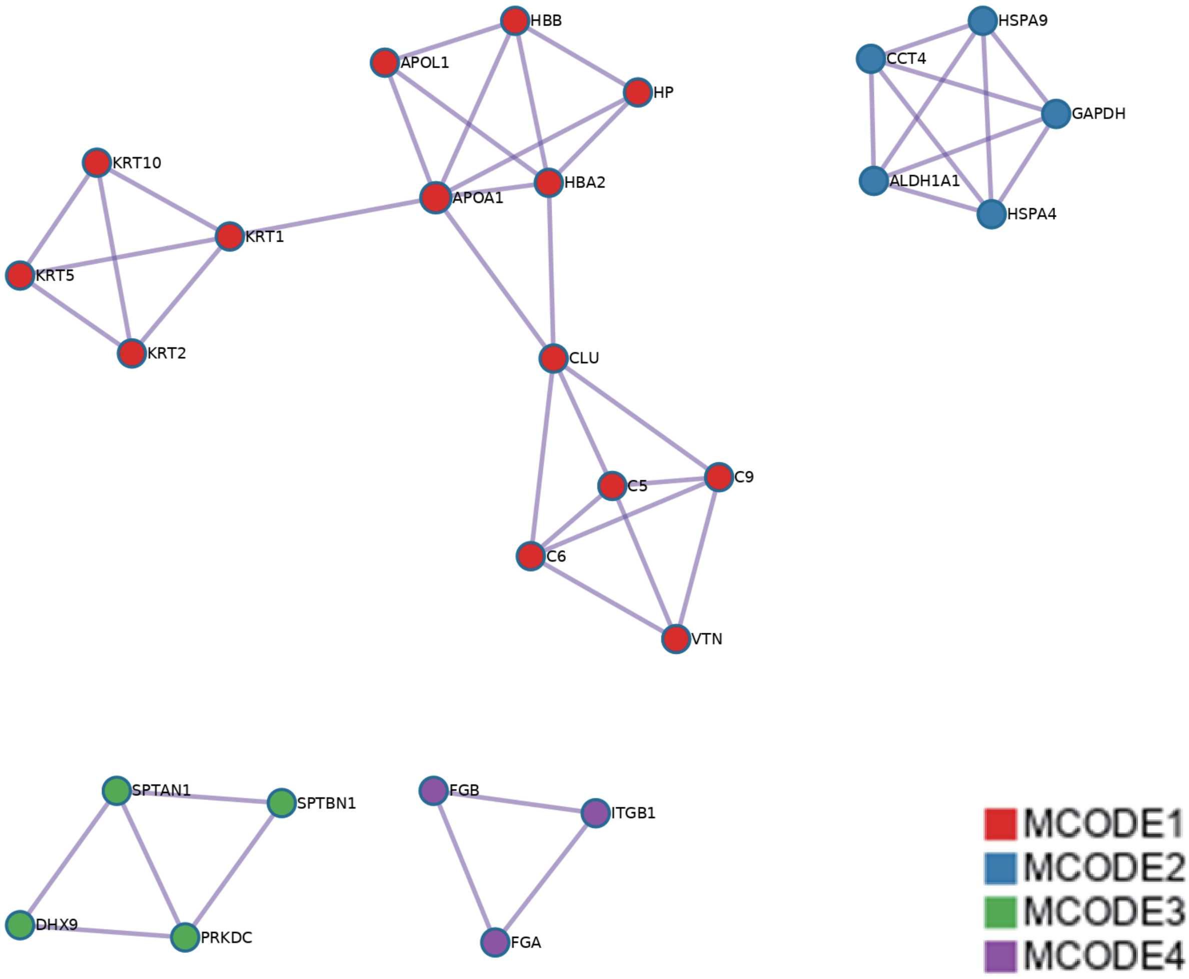

Supplement: Supplementary Figure 1 — PPI analyses of differentially expressed proteins in IBC vs cancer-adjacent and normal breast tissues using Metascape. (A) PPI network of proteins encoded by differentially expressed proteins. (B) Modules selected from PPI network using MCODE. Nodes represent differentially expressed proteins; lines represent interaction relationships between nodes. (C) Independent functional enrichment analysis of MCODE components. [file Image_1.pdf]
