# Supplementary material for: Isobaric Tags for Relative and Absolute Quantitation in Proteomic Analysis of Potential Biomarkers in Invasive Cancer, Ductal Carcinoma In Situ, and Mammary Fibroadenoma
Source: Front Oncol. 2020 Oct 21;10:574552. doi: 10.3389/fonc.2020.574552 (PMC7640741; doi:10.3389/fonc.2020.574552)
Supplement: Supplementary Figure 2 — PPI analyses of differentially expressed proteins in DCIS vs cancer-adjacent and normal breast tissues using Metascape. (A) PPI network of proteins encoded by differentially expressed proteins. (B) Modules selected from PPI network using MCODE. Nodes represent differentially expressed proteins; lines represent interaction relationships between nodes. (C) Independent functional enrichment analysis of MCODE components. [file Image_2.pdf]

a

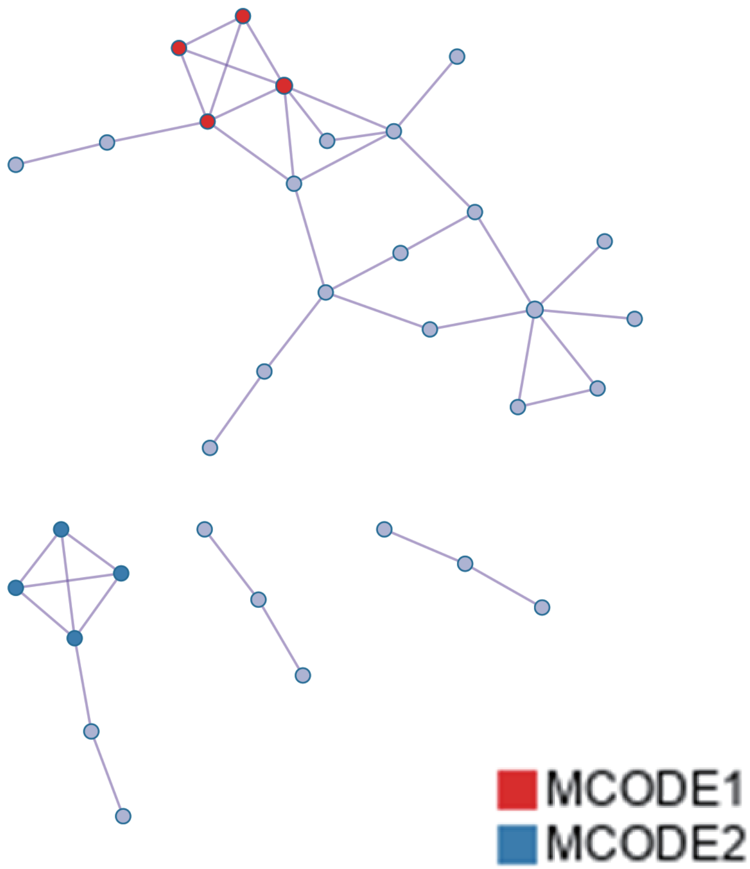

b

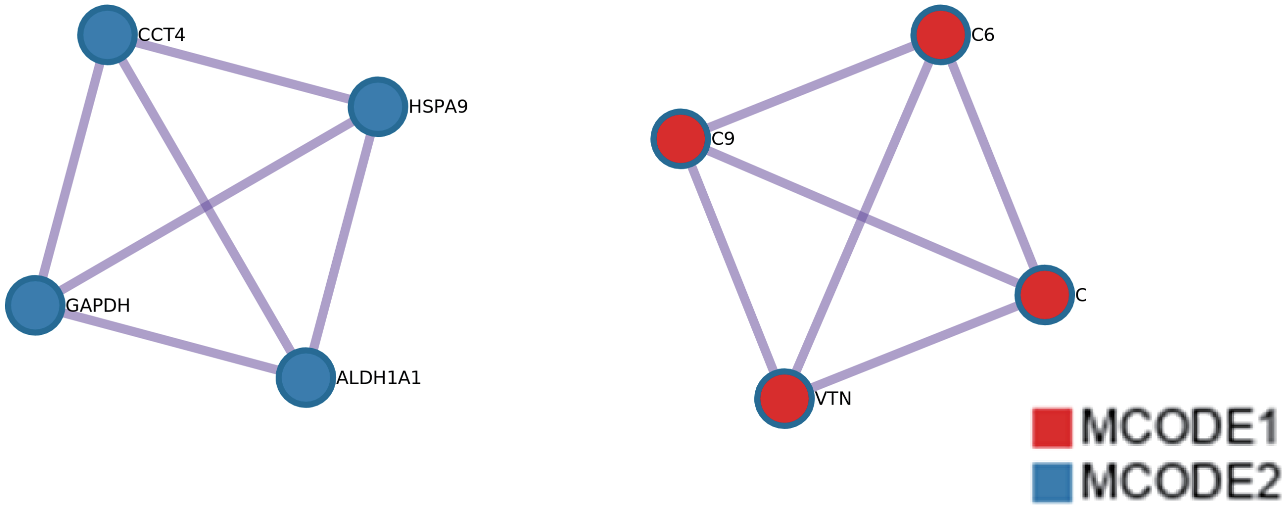

c

Color	MCODE	GO	Description	Log10(P)
	MCODE_1	R-HSA-977606	Regulation of Complement cascade	-10.9
	MCODE_1	R-HSA-166658	Complement cascade	-10.5
	MCODE_1	R-HSA-166665	Terminal pathway of complement	-10.0
