# Supplementary material for: Isobaric Tags for Relative and Absolute Quantitation in Proteomic Analysis of Potential Biomarkers in Invasive Cancer, Ductal Carcinoma In Situ, and Mammary Fibroadenoma
Source: Front Oncol. 2020 Oct 21;10:574552. doi: 10.3389/fonc.2020.574552 (PMC7640741; doi:10.3389/fonc.2020.574552)
Supplement: Supplementary Figure 3 — PPI analyses of differentially expressed proteins associated with growing level of malignancy in IBC and DCIS tissues using Metascape. (A) PPI network of proteins encoded by differentially expressed proteins. (B) Modules selected from PPI network using MCODE. Nodes represent differentially expressed proteins; lines represent interaction relationships between nodes. (C) Independent functional enrichment analysis of MCODE components. [file Image_3.pdf]

a

b

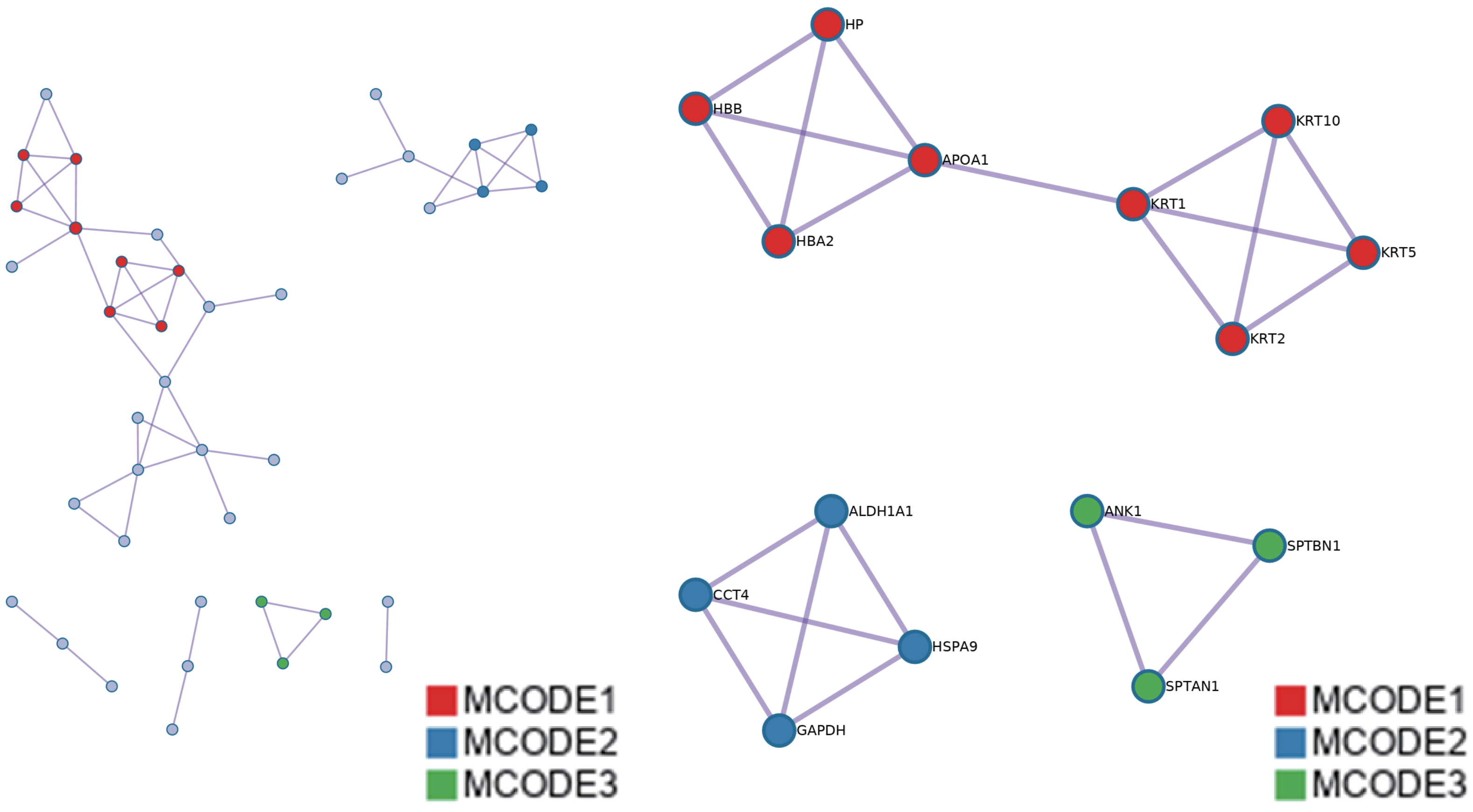

c

Color	MCODE	GO	Description	Log10(P)
(red)	MCODE_1	R-HSA-2168880	Scavenging of heme from plasma	-11.5
(red)	MCODE_1	R-HSA-2173782	Binding and Uptake of Ligands by Scavenger Receptors	-9.3
(red)	MCODE_1	GO:0070268	cornification	-7.5
(green)	MCODE_3	R-HSA-445095	Interaction between L1 and Ankyrins	-8.7
(green)	MCODE_3	R-HSA-6807878	COPI-mediated anterograde transport	-7.1
(green)	MCODE_3	R-HSA-373760	L1CAM interactions	-6.9
