# Supplementary material for: Isobaric Tags for Relative and Absolute Quantitation in Proteomic Analysis of Potential Biomarkers in Invasive Cancer, Ductal Carcinoma In Situ, and Mammary Fibroadenoma
Source: Front Oncol. 2020 Oct 21;10:574552. doi: 10.3389/fonc.2020.574552 (PMC7640741; doi:10.3389/fonc.2020.574552)
Supplement: Supplementary Table 1 — 20 up-regulated proteins of IBC tissues compared to adjacent and normal tissues. Differentially expressed proteins with ≥2-fold higher differences in IBC compared to both IBC-adjacent and normal tissues were screened. [file Table_1.docx]

**Table 1: 20 up-regulated proteins of IBC tissues compared to adjacent and normal tissues**

| **Accession** | **Name** | **Sequence coverage (%)** | **Peptides (95%)** |
| --- | --- | --- | --- |
| tr\|E5KRG5\|E5KRG5_HUMAN | hCG | 50.41 | 17 |
| sp\|P49411\|EFTU_HUMAN | TUFM | 54.2 | 19 |
| sp\|P34932\|HSP74_HUMAN | HSPA4 | 50.24 | 23 |
| tr\|A7BI36\|A7BI36_HUMAN | RRBP1 | 63.64 | 27 |
| tr\|Q8N1C8\|Q8N1C8_HUMAN | HSPA9 | 70.19 | 33 |
| sp\|P23396\|RS3_HUMAN | RPS3 | 57.61 | 12 |
| tr\|A8K4W6\|A8K4W6_HUMAN | PGK1 | 88.25 | 58 |
| sp\|P78527-2\|PRKDC_HUMAN | PRKDC | 29.75 | 32 |
| sp\|P04406\|G3P_HUMAN | GAPDH | 91.94 | 134 |
| tr\|D6RGG3\|D6RGG3_HUMAN | COL12A1 | 45.56 | 85 |
| tr\|Q6IAT1\|Q6IAT1_HUMAN | GDI2 | 59.78 | 30 |
| sp\|Q9NSE4\|SYIM_HUMAN | IARS2 | 29.55 | 11 |
| sp\|Q9HB40\|RISC_HUMAN | SCPEP1 | 25.22 | 4 |
| sp\|Q08211\|DHX9_HUMAN | DHX9 | 40.31 | 26 |
| sp\|P50991\|TCPD_HUMAN | CCT4 | 43.78 | 12 |
| sp\|P50454\|SERPH_HUMAN | SERPINH1 | 70.81 | 25 |
| tr\|Q53HF3\|Q53HF3_HUMAN | GLA | 26.34 | 3 |
| sp\|O60701\|UGDH_HUMAN | UGDH | 55.67 | 15 |
| sp\|P43490\|NAMPT_HUMAN | NAMPT | 42.36 | 10 |
| sp\|P24347\|MMP11_HUMAN | MMP11 | 26.02 | 1 |
