# Supplementary material for: Isobaric Tags for Relative and Absolute Quantitation in Proteomic Analysis of Potential Biomarkers in Invasive Cancer, Ductal Carcinoma In Situ, and Mammary Fibroadenoma
Source: Front Oncol. 2020 Oct 21;10:574552. doi: 10.3389/fonc.2020.574552 (PMC7640741; doi:10.3389/fonc.2020.574552)
Supplement: Supplementary Table 3 — Step-changing of 7 up-regulated and 11 down-regulated proteins in IBC, adjacent and normal tissues. Differentially expressed proteins with ≥2-fold (higher or lower) differences in IBC or IBC-adjacent tissues compared to normal tissues were screened (P<0.05). Next, proteins with higher or lower differences in IBC compared to IBC-adjacent tissues were further screened. [file Table_3.docx]

**Table 3: Step-changing of 7 up-regulated and 11 down-regulated proteins in IBC, adjacent and normal tissues**

|  | **Accession** | **Name** | **Sequence coverage (%)** | **Peptides (95%)** |
| --- | --- | --- | --- | --- |
| Up | sp\|P23368\|MAOM_HUMAN | ME2 | 15.41 | 2 |
|  | sp\|P21333-2\|FLNA_HUMAN | FLNA | 75.03 | 236 |
|  | sp\|Q15149-3\|PLEC_HUMAN | PLEC | 58.45 | 122 |
|  | sp\|Q9HB40\|RISC_HUMAN | SCPEP1 | 25.22 | 4 |
|  | tr\|Q53HF3\|Q53HF3_HUMAN | GLA | 26.34 | 3 |
|  | tr\|Q2TB59\|Q2TB59_HUMAN | NNT | 19.52 | 4 |
|  | sp\|P24347\|MMP11_HUMAN | MMP11 | 26.02 | 1 |
|  |  |  |  |  |
| Down | sp\|P13645\|K1C10_HUMAN | KRT10 | 57.88 | 42 |
|  | tr\|H6VRG2\|H6VRG2_HUMAN | KRT1 | 56.21 | 45 |
|  | sp\|P35908\|K22E_HUMAN | KRT2 | 54.62 | 26 |
|  | tr\|B2R853\|B2R853_HUMAN | KRT6E | 68.26 | 58 |
|  | sp\|P51884\|LUM_HUMAN | LUM | 68.34 | 101 |
|  | tr\|D1MGQ2\|D1MGQ2_HUMAN | HBA2 | 99.3 | 288 |
|  | tr\|D1MGQ2\|D1MGQ2_HUMAN | CA1 | 70.88 | 26 |
|  | tr\|D9YZU5\|D9YZU5_HUMAN | HBB | 96.6 | 429 |
|  | sp\|P32119\|PRDX2_HUMAN | PRDX2 | 80.3 | 30 |
|  | tr\|Q5U0J2\|Q5U0J2_HUMAN | CSRP1 | 67.88 | 21 |
|  | sp\|Q05682-5\|CALD1_HUMAN | CALD1 | 65.23 | 39 |
