# Supplementary material for: Isobaric Tags for Relative and Absolute Quantitation in Proteomic Analysis of Potential Biomarkers in Invasive Cancer, Ductal Carcinoma In Situ, and Mammary Fibroadenoma
Source: Front Oncol. 2020 Oct 21;10:574552. doi: 10.3389/fonc.2020.574552 (PMC7640741; doi:10.3389/fonc.2020.574552)
Supplement: Supplementary Table 4 — 4 up-regulated proteins of DCIS tissues compared to adjacent and normal tissues. Differentially expressed proteins with ≥2-fold higher differences in DCIS compared to both DCIS-adjacent and normal tissues were screened. [file Table_4.docx]

**Table 4: 4 up-regulated proteins of DCIS tissues compared to adjacent and normal tissues**

| **Accession** | **Name** | **Sequence coverage (%)** | **Peptides (95%)** |
| --- | --- | --- | --- |
| sp\|P04406\|G3P_HUMAN | GAPDH | 50.41 | 91.94 |
| tr\|Q8N1C8\|Q8N1C8_HUMAN | HSPA9 | 50.24 | 70.19 |
| sp\|P50991\|TCPD_HUMAN | CCT4 | 91.94 | 43.78 |
| sp\|Q9HB40\|RISC_HUMAN | SCPEP1 | 59.78 | 25.22 |
