# Supplementary material for: Isobaric Tags for Relative and Absolute Quantitation in Proteomic Analysis of Potential Biomarkers in Invasive Cancer, Ductal Carcinoma In Situ, and Mammary Fibroadenoma
Source: Front Oncol. 2020 Oct 21;10:574552. doi: 10.3389/fonc.2020.574552 (PMC7640741; doi:10.3389/fonc.2020.574552)
Supplement: Supplementary Table 5 — 48 down-regulated proteins of DCIS tissues compared to adjacent and normal tissues. Differentially expressed proteins with ≥2-fold lower differences in DCIS compared to both DCIS-adjacent and normal tissues were screened. [file Table_5.docx]

**Table 5: 48 down-regulated proteins of DCIS tissues compared to adjacent and normal tissues**

| **Accession** | **Name** | **Sequence coverage (%)** | **Peptides (95%)** |
| --- | --- | --- | --- |
| sp\|P13645\|K1C10_HUMAN | KRT10 | 57.88 | 42 |
| tr\|A8KAM5\|A8KAM5_HUMAN | SFRP1 | 42.81 | 8 |
| tr\|B2R582\|B2R582_HUMAN | CLEC3B | 54.46 | 13 |
| sp\|Q07507\|DERM_HUMAN | DPT | 54.23 | 11 |
| sp\|P02647\|APOA1_HUMAN | APOA1 | 85.02 | 135 |
| sp\|P51884\|LUM_HUMAN | LUM | 68.34 | 101 |
| tr\|D6RAK8\|D6RAK8_HUMAN | GC | 82.35 | 77 |
| tr\|Q6FH10\|Q6FH10_HUMAN | DCN | 77.16 | 58 |
| tr\|A5PL27\|A5PL27_HUMAN | CP | 62.25 | 65 |
| tr\|B2R8I2\|B2R8I2_HUMAN | HRG | 46.1 | 26 |
| sp\|Q9BX66-12\|SRBS1_HUMAN | SORBS1 | 29.75 | 5 |
| sp\|P02790\|HEMO_HUMAN | HPX | 79.87 | 116 |
| sp\|P12111-2\|CO6A3_HUMAN | COL6A3 | 64.39 | 234 |
| tr\|G3V5I3\|G3V5I3_HUMAN | SERPINA3 | 59.82 | 34 |
| sp\|P12109\|CO6A1_HUMAN | COL6A1 | 60.7 | 92 |
| sp\|P01591\|IGJ_HUMAN | IGJ | 62.26 | 18 |
| sp\|P35527\|K1C9_HUMAN | KRT9 | 37.72 | 11 |
| sp\|P08294\|SODE_HUMAN | SOD3 | 57.5 | 19 |
| sp\|P01031\|CO5_HUMAN | C5 | 22.97 | 8 |
| tr\|A4D2D2\|A4D2D2_HUMAN | PCOLCE | 47.44 | 15 |
| sp\|P06727\|APOA4_HUMAN | APOA4 | 77.02 | 30 |
| tr\|B2R7F8\|B2R7F8_HUMAN | PLG | 61.85 | 32 |
| tr\|B0V046\|B0V046_HUMAN | TNXB | 24.92 | 19 |
| sp\|P00734\|THRB_HUMAN | F2 | 63.18 | 50 |
| tr\|D9ZGG2\|D9ZGG2_HUMAN | VTN | 52.3 | 17 |
| tr\|B4E1C2\|B4E1C2_HUMAN | KNG1 | 48.91 | 30 |
| sp\|P01008\|ANT3_HUMAN | SERPINC1 | 54.31 | 43 |
| sp\|P02748\|CO9_HUMAN | C9 | 39.53 | 18 |
| tr\|E7EVA3\|E7EVA3_HUMAN | CFB | 50.32 | 55 |
| sp\|P02763\|A1AG1_HUMAN | ORM1 | 67.66 | 44 |
| tr\|G8JLA8\|G8JLA8_HUMAN | TGFBI | 53.88 | 31 |
| tr\|H0YGH4\|H0YGH4_HUMAN | A2M | 65.41 | 101 |
| tr\|Q8IVC0\|Q8IVC0_HUMAN | SERPIND1 | 31.66 | 13 |
| sp\|P08185\|CBG_HUMAN | SERPINA6 | 29.38 | 9 |
| sp\|P04217\|A1BG_HUMAN | A1BG | 61.82 | 36 |
| tr\|A8K3E4\|A8K3E4_HUMAN | FGA | 63.2 | 63 |
| sp\|P00738\|HPT_HUMAN | HP | 88.67 | 69 |
| sp\|P13671\|CO6_HUMAN | C6 | 29.76 | 9 |
| sp\|O60240\|PLIN1_HUMAN | PLIN1 | 49.62 | 15 |
| tr\|Q6FHG6\|Q6FHG6_HUMAN | PRELP | 54.19 | 32 |
| sp\|P02545\|LMNA_HUMAN | LMNA | 73.8 | 63 |
| sp\|P02760\|AMBP_HUMAN | AMBP | 45.45 | 13 |
| tr\|Q5U0J2\|Q5U0J2_HUMAN | CSRP1 | 67.88 | 21 |
| sp\|P08603\|CFAH_HUMAN | CFH | 57.76 | 59 |
| sp\|P00352\|AL1A1_HUMAN | ALDH1A1 | 69.06 | 33 |
| tr\|C0JYY2\|C0JYY2_HUMAN | APOB | 39.71 | 84 |
| sp\|Q13813-2\|SPTA2_HUMAN | SPTAN1 | 60.07 | 89 |
| tr\|Q53FI7\|Q53FI7_HUMAN | FHL1 | 60 | 10 |
