# Supplementary material for: Isobaric Tags for Relative and Absolute Quantitation in Proteomic Analysis of Potential Biomarkers in Invasive Cancer, Ductal Carcinoma In Situ, and Mammary Fibroadenoma
Source: Front Oncol. 2020 Oct 21;10:574552. doi: 10.3389/fonc.2020.574552 (PMC7640741; doi:10.3389/fonc.2020.574552)
Supplement: Supplementary Table 6 — Step-changing of 6 up-regulated and 8 down-regulated proteins in DCIS, adjacent and normal tissues. Differentially expressed proteins with ≥2-fold (higher or lower) differences in DCIS or DCIS-adjacent tissues compared to normal tissues were screened (P<0.05). Next, proteins with higher or lower differences in DCIS compared to DCIS-adjacent tissues were further screened. [file Table_6.docx]

**Table 6: Step-changing of 6 up-regulated and 8 down-regulated proteins in DCIS, adjacent and normal tissues**

|  | **Accession** | **Name** | **Sequence coverage (%)** | **Peptides (95%)** |
| --- | --- | --- | --- | --- |
| Up | tr\|B2RAY1\|B2RAY1_HUMAN | STAM | 22.59 | 2 |
|  | tr\|A4D0Z3\|A4D0Z3_HUMAN | ARF5 | 75.56 | 23 |
|  | sp\|P08133\|ANXA6_HUMAN | ANXA6 | 68.05 | 49 |
|  | sp\|Q9HB40\|RISC_HUMAN | SCPEP1 | 25.22 | 4 |
|  | sp\|P23368\|MAOM_HUMAN | ME2 | 15.41 | 2 |
|  | sp\|O76024\|WFS1_HUMAN | WFS1 | 22.25 | 2 |
|  |  |  |  |  |
| Down | tr\|H6VRG2\|H6VRG2_HUMAN | KRT1 | 56.21 | 45 |
|  | sp\|P13645\|K1C10_HUMAN | KRT10 | 57.88 | 42 |
|  | tr\|B2R853\|B2R853_HUMAN | KRT6E | 68.26 | 58 |
|  | sp\|P02647\|APOA1_HUMAN | APOA1 | 85.02 | 135 |
|  | sp\|P15924\|DESP_HUMAN | DSP | 35.88 | 18 |
|  | sp\|P51884\|LUM_HUMAN | LUM | 68.34 | 101 |
|  | sp\|P16157-7\|ANK1_HUMAN | ANK1 | 32.42 | 14 |
|  | sp\|P00734\|THRB_HUMAN | F2 | 63.18 | 50 |
