# Supplementary material for: Isobaric Tags for Relative and Absolute Quantitation in Proteomic Analysis of Potential Biomarkers in Invasive Cancer, Ductal Carcinoma In Situ, and Mammary Fibroadenoma
Source: Front Oncol. 2020 Oct 21;10:574552. doi: 10.3389/fonc.2020.574552 (PMC7640741; doi:10.3389/fonc.2020.574552)
Supplement: Supplementary Table 7 — 6 up-regulated proteins of fibroadenoma tissues compared to adjacent and normal tissues. Differentially expressed proteins with ≥2-fold higher differences in fibroadenoma compared to both fibroadenoma-adjacent and normal tissues were screened. [file Table_7.docx]

**Table 7: 6 up-regulated proteins of fibroadenoma tissues compared to adjacent and normal tissues**

| **Accession** | **Name** | **Sequence coverage (%)** | **Peptides (95%)** |
| --- | --- | --- | --- |
| sp\|P08133\|ANXA6_HUMAN | ANXA6 | 68.05 | 49 |
| sp\|P55072\|TERA_HUMAN | VCP | 62.41 | 36 |
| sp\|P50454\|SERPH_HUMAN | SERPINH1 | 70.81 | 25 |
| tr\|Q53HF3\|Q53HF3_HUMAN | Galactosidase alpha | 26.34 | 3 |
| tr\|Q2TB59\|Q2TB59_HUMAN | NNT | 19.52 | 4 |
| sp\|P24347\|MMP11_HUMAN | MMP11 | 26.02 | 1 |
