# Supplementary material for: Isobaric Tags for Relative and Absolute Quantitation in Proteomic Analysis of Potential Biomarkers in Invasive Cancer, Ductal Carcinoma In Situ, and Mammary Fibroadenoma
Source: Front Oncol. 2020 Oct 21;10:574552. doi: 10.3389/fonc.2020.574552 (PMC7640741; doi:10.3389/fonc.2020.574552)
Supplement: Supplementary Table 8 — 38 down-regulated proteins of fibroadenoma tissues compared to adjacent and normal tissue. Differentially expressed proteins with ≥2-fold lower differences in fibroadenoma compared to both fibroadenoma-adjacent and normal tissues were screened. [file Table_8.docx]

**Table 8: 38 down-regulated proteins of fibroadenoma tissues compared to adjacent and normal tissues**

| **Accession** | **Name** | **Sequence coverage (%)** | **Peptides (95%)** |
| --- | --- | --- | --- |
| sp\|P13645\|K1C10_HUMAN | KRT10 | 57.88 | 42 |
| tr\|H6VRG2\|H6VRG2_HUMAN | KRT1 | 56.21 | 45 |
| tr\|F8W1L5\|F8W1L5_HUMAN | GPD1 | 46.32 | 8 |
| sp\|Q16853\|AOC3_HUMAN | AOC3 | 36.17 | 25 |
| tr\|Q6FHG6\|Q6FHG6_HUMAN | PRELP | 54.19 | 32 |
| tr\|Q6FH10\|Q6FH10_HUMAN | DCN | 77.16 | 58 |
| sp\|P08294\|SODE_HUMAN | SOD3 | 57.5 | 19 |
| tr\|H0YGH4\|H0YGH4_HUMAN | A2M | 65.41 | 101 |
| sp\|P00915\|CAH1_HUMAN | CA1 | 70.88 | 26 |
| tr\|D9YZU5\|D9YZU5_HUMAN | HBB | 96.6 | 429 |
| sp\|O60240\|PLIN1_HUMAN | PLIN1 | 49.62 | 15 |
| sp\|P16157-7\|ANK1_HUMAN | ANK1 | 32.42 | 14 |
| sp\|Q07507\|DERM_HUMAN | DPT | 54.23 | 11 |
| tr\|C0JYY2\|C0JYY2_HUMAN | APOB | 39.71 | 84 |
| sp\|P13671\|CO6_HUMAN | C6 | 29.76 | 9 |
| sp\|P04217\|A1BG_HUMAN | A1BG | 61.82 | 36 |
| tr\|Q5VVQ8\|Q5VVQ8_HUMAN | C4BPA | 34.51 | 9 |
| tr\|A5PL27\|A5PL27_HUMAN | CP | 62.25 | 65 |
| tr\|A8K3E4\|A8K3E4_HUMAN | FGA | 63.2 | 63 |
| sp\|P51884\|LUM_HUMAN | LUM | 68.34 | 101 |
| tr\|B2RMS9\|B2RMS9_HUMAN | ITIH4 | 43.76 | 33 |
| sp\|P06727\|APOA4_HUMAN | APOA4 | 77.02 | 30 |
| sp\|P12109\|CO6A1_HUMAN | COL6A1 | 60.7 | 92 |
| sp\|P04040\|CATA_HUMAN | CAT | 52.18 | 17 |
| tr\|D6RAK8\|D6RAK8_HUMAN | GC | 82.35 | 77 |
| sp\|P00352\|AL1A1_HUMAN | ALDH1A1 | 69.06 | 33 |
| tr\|B4E1C2\|B4E1C2_HUMAN | KNG1 | 48.91 | 30 |
| sp\|P08603\|CFAH_HUMAN | CFH | 57.76 | 59 |
| tr\|Q8IVC0\|Q8IVC0_HUMAN | SERPIND1 | 31.66 | 13 |
| sp\|P02675\|FIBB_HUMAN | FGB | 78.21 | 78 |
| tr\|D9IWP9\|D9IWP9_HUMAN | B2G1 | 78.83 | 33 |
| sp\|Q16610\|ECM1_HUMAN | ECM1 | 36.3 | 7 |
| tr\|A4D2D2\|A4D2D2_HUMAN | PCOLCE | 47.44 | 15 |
| sp\|P10909-2\|CLUS_HUMAN | CLU | 48.9 | 26 |
| sp\|P00738\|HPT_HUMAN | HP | 88.67 | 69 |
| sp\|P02760\|AMBP_HUMAN | AMBP | 45.45 | 13 |
| sp\|Q96Q06-2\|PLIN4_HUMAN | PLIN4 | 57.91 | 42 |
| tr\|B0V046\|B0V046_HUMAN | TNXB | 24.92 | 19 |
