# Supplementary material for: Isobaric Tags for Relative and Absolute Quantitation in Proteomic Analysis of Potential Biomarkers in Invasive Cancer, Ductal Carcinoma In Situ, and Mammary Fibroadenoma
Source: Front Oncol. 2020 Oct 21;10:574552. doi: 10.3389/fonc.2020.574552 (PMC7640741; doi:10.3389/fonc.2020.574552)
Supplement: Supplementary Table 9 — Step-changing of 6 up-regulated and 7 down-regulated proteins in fibroadenoma, adjacent and normal tissues. Differentially expressed proteins with ≥2-fold (higher or lower) differences in fibroadenoma or fibroadenoma-adjacent tissues compared to normal tissues were screened (P<0.05). Next, proteins with higher or lower differences in fibroadenoma compared to fibroadenoma-adjacent tissues were further screened. [file Table_9.docx]

**Table 9: Step-changing of 6 up-regulated and 7 down-regulated proteins in fibroadenoma, adjacent and normal tissues**

|  | **Accession** | **Name** | **Sequence coverage (%)** | **Peptides (95%)** |
| --- | --- | --- | --- | --- |
| Up | sp\|A8MXV4\|NUD19_HUMAN | NUDT19 | 29.33 | 2 |
|  | sp\|P35555\|FBN1_HUMAN | FBN1 | 42.46 | 50 |
|  | tr\|A8K525\|A8K525_HUMAN | NONO | 51.38 | 10 |
|  | sp\|P21333-2\|FLNA_HUMAN | FLNA | 75.03 | 236 |
|  | sp\|Q9HB40\|RISC_HUMAN | SCPEP1 | 25.22 | 4 |
|  | tr\|Q53HF3\|Q53HF3_HUMAN | Galactosidase alpha | 26.34 | 3 |
|  |  |  |  |  |
| Down | sp\|P13645\|K1C10_HUMAN | KRT10 | 57.88 | 42 |
|  | tr\|H6VRG2\|H6VRG2_HUMAN | KRT1 | 56.21 | 45 |
|  | tr\|B2R853\|B2R853_HUMAN | KRT6E | 68.26 | 58 |
|  | sp\|O60240\|PLIN1_HUMAN | PLIN1 | 49.62 | 15 |
|  | tr\|D1MGQ2\|D1MGQ2_HUMAN | HBA2 | 99.3 | 288 |
|  | tr\|Q53FI7\|Q53FI7_HUMAN | FHL1 | 60 | 10 |
|  | sp\|P23368\|MAOM_HUMAN | ME2 | 15.41 | 2 |
