# Supplementary material for: Isobaric Tags for Relative and Absolute Quantitation in Proteomic Analysis of Potential Biomarkers in Invasive Cancer, Ductal Carcinoma In Situ, and Mammary Fibroadenoma
Source: Front Oncol. 2020 Oct 21;10:574552. doi: 10.3389/fonc.2020.574552 (PMC7640741; doi:10.3389/fonc.2020.574552)
Supplement: Supplementary Table 10 — Specific up- or down-regulated proteins in IBC, DCIS and fibroadenoma tissues. Differentially expressed proteins with ≥2-fold (higher or lower) differences in IBC/DCIS/fibroadenoma compared to matched tumor-adjacent tissues and normal tissues were screened. Intersections of upregulated and downregulated proteins for IBC, DCIS, and fibroadenoma tissues were determined using Wayne chart way. [file Table_10.docx]

**Table 10: Specific up- or down-regulated proteins in IBC, DCIS and fibroadenoma tissues**

|  | **Up-regulated** | **Down-regulated** |
| --- | --- | --- |
| **IBC** | hCG, TUFM, HSPA4, RRBP1, RPS3, PGK1, PRKDC, COL12A1, GDI2, IARS2, DHX9, GLA, UGDH, NAMPT. | PIP, APOD, KRT2, APOA2, KRT6E, IL16, AZGP1,  HBA2, KRT5, HBG1, ITIH2, SPTBN1, COL18A1,  SERPINA4, PEBP1, APOL1, GGT5, MAOB, ITGB4,  EHD2, APCS, ITGB1, PTN. |
| **DCIS** | None | SFRP1, KRT9, TGFBI, CSRP1 |
| **Fibroadenoma** | ANXA6, VCP, Galactosidase alpha, NNT | None |
