# Supplementary material for: Isobaric Tags for Relative and Absolute Quantitation in Proteomic Analysis of Potential Biomarkers in Invasive Cancer, Ductal Carcinoma In Situ, and Mammary Fibroadenoma
Source: Front Oncol. 2020 Oct 21;10:574552. doi: 10.3389/fonc.2020.574552 (PMC7640741; doi:10.3389/fonc.2020.574552)
Supplement: Supplementary Table 11 — 10 up-regulated and 55 down-regulated proteins with growing level of malignancy in IBC and DCIS tissues. Differentially expressed proteins with ≥2-fold (higher or lower) differences in IBC or DCIS tissues compared to normal tissues were screened (P<0.05). Next, proteins with higher or lower differences in IBC compared to DCIS tissues were further screened. [file Table_11.docx]

**Table11: 10 up-regulated and 55 down-regulated proteins with growing level of malignancy in IBC and DCIS tissues**

|  | **Accession** | **Name** | **Sequence coverage (%)** | **Peptides (95%)** |
| --- | --- | --- | --- | --- |
| Up | tr\|A7BI36\|A7BI36_HUMAN | RRBP1 | 63.64 | 27 |
|  | tr\|Q8N1C8\|Q8N1C8_HUMAN | HSPA9 | 70.19 | 33 |
|  | tr\|A8K4W6\|A8K4W6_HUMAN | PGK1 | 88.25 | 58 |
|  | sp\|P04406\|G3P_HUMAN | GAPDH | 91.94 | 134 |
|  | sp\|Q9NSE4\|SYIM_HUMAN | IARS2 | 29.55 | 11 |
|  | sp\|P50991\|TCPD_HUMAN | CCT4 | 43.78 | 12 |
|  | tr\|Q53HF3\|Q53HF3_HUMAN | Galactosidase alpha | 26.34 | 3 |
|  | sp\|P43490\|NAMPT_HUMAN | NAMPT | 42.36 | 10 |
|  | tr\|Q2TB59\|Q2TB59_HUMAN | NNT | 19.52 | 4 |
|  | sp\|O76024\|WFS1_HUMAN | WFS1 | 22.25 | 2 |
|  |  |  |  |  |
|  |  |  |  |  |
| Down | sp\|P13645\|K1C10_HUMAN | KRT10 | 57.88 | 42 |
|  | tr\|H6VRG2\|H6VRG2_HUMAN | KRT1 | 56.21 | 45 |
|  | sp\|P12273\|PIP_HUMAN | PIP | 85.62 | 31 |
|  | sp\|P35908\|K22E_HUMAN | KRT2 | 54.62 | 26 |
|  | tr\|Q6FH10\|Q6FH10_HUMAN | DCN | 77.16 | 58 |
|  | tr\|B2R853\|B2R853_HUMAN | KRT6E | 68.26 | 58 |
|  | sp\|P02760\|AMBP_HUMAN | AMBP | 45.45 | 13 |
|  | sp\|P02647\|APOA1_HUMAN | APOA1 | 85.02 | 135 |
|  | tr\|D6RAK8\|D6RAK8_HUMAN | GC | 82.35 | 77 |
|  | tr\|H0YGH4\|H0YGH4_HUMAN | A2M | 65.41 | 101 |
|  | tr\|D9ZGG2\|D9ZGG2_HUMAN | VTN | 52.3 | 17 |
|  | sp\|P51884\|LUM_HUMAN | LUM | 68.34 | 101 |
|  | sp\|P06727\|APOA4_HUMAN | APOA4 | 77.02 | 30 |
|  | tr\|B4E1C2\|B4E1C2_HUMAN | KNG1 | 48.91 | 30 |
|  | sp\|P08294\|SODE_HUMAN | SOD3 | 57.5 | 19 |
|  | tr\|A5PL27\|A5PL27_HUMAN | CP | 62.25 | 65 |
|  | sp\|P12109\|CO6A1_HUMAN | COL6A1 | 60.7 | 92 |
|  | tr\|D1MGQ2\|D1MGQ2_HUMAN | HBA2 | 99.3 | 288 |
|  | sp\|P16157-7\|ANK1_HUMAN | ANK1 | 32.42 | 14 |
|  | sp\|P01008\|ANT3_HUMAN | SERPINC1 | 54.31 | 43 |
|  | sp\|P13671\|CO6_HUMAN | C6 | 29.76 | 9 |
|  | sp\|P02675\|FIBB_HUMAN | FGB | 78.21 | 78 |
|  | sp\|P04217\|A1BG_HUMAN | A1BG | 61.82 | 36 |
|  | tr\|E7EVA3\|E7EVA3_HUMAN | CFB | 50.32 | 55 |
|  | sp\|P02790\|HEMO_HUMAN | HPX | 79.87 | 116 |
|  | tr\|G3V5I3\|G3V5I3_HUMAN | SERPINA3 | 59.82 | 34 |
|  | sp\|Q13813-2\|SPTA2_HUMAN | SPTAN1 | 60.07 | 89 |
|  | sp\|P08603\|CFAH_HUMAN | CFH | 57.76 | 59 |
|  | tr\|B2RMS9\|B2RMS9_HUMAN | ITIH4 | 43.76 | 33 |
|  | tr\|Q5VVQ8\|Q5VVQ8_HUMAN | C4BPA | 34.51 | 9 |
|  | sp\|P12111-2\|CO6A3_HUMAN | COL6A3 | 64.39 | 234 |
|  | tr\|B2R8I2\|B2R8I2_HUMAN | HRG | 46.1 | 26 |
|  | tr\|B2R582\|B2R582_HUMAN | CLEC3B | 54.46 | 13 |
|  | sp\|P13647\|K2C5_HUMAN | KRT5 | 56.61 | 34 |
|  | tr\|D9YZU5\|D9YZU5_HUMAN | HBB | 96.6 | 429 |
|  | sp\|P00734\|THRB_HUMAN | F2 | 63.18 | 50 |
|  | sp\|P01591\|IGJ_HUMAN | IGJ | 62.26 | 18 |
|  | sp\|P00738\|HPT_HUMAN | HP | 88.67 | 69 |
|  | sp\|P02763\|A1AG1_HUMAN | ORM1 | 67.66 | 44 |
|  | sp\|P04040\|CATA_HUMAN | CAT | 52.18 | 17 |
|  | sp\|P00352\|AL1A1_HUMAN | ALDH1A1 | 69.06 | 33 |
|  | tr\|B2ZZ89\|B2ZZ89_HUMAN | SPTBN1 | 54.78 | 74 |
|  | tr\|Q6FHG6\|Q6FHG6_HUMAN | PRELP | 54.19 | 32 |
|  | tr\|B2R7F8\|B2R7F8_HUMAN | PLG | 61.85 | 32 |
|  | sp\|Q16853\|AOC3_HUMAN | AOC3 | 36.17 | 25 |
|  | sp\|P02748\|CO9_HUMAN | C9 | 39.53 | 18 |
|  | sp\|O60240\|PLIN1_HUMAN | PLIN1 | 49.62 | 15 |
|  | tr\|Q53FI7\|Q53FI7_HUMAN | FHL1 | 60 | 10 |
|  | tr\|B0V046\|B0V046_HUMAN | TNXB | 24.92 | 19 |
|  | tr\|Q8IVC0\|Q8IVC0_HUMAN | SERPIND1 | 31.66 | 13 |
|  | sp\|P08185\|CBG_HUMAN | SERPINA6 | 29.38 | 9 |
|  | tr\|B2R815\|B2R815_HUMAN | SERPINA4 | 34.89 | 6 |
|  | tr\|Q5U0J2\|Q5U0J2_HUMAN | CSRP1 | 67.88 | 21 |
|  | sp\|P02545\|LMNA_HUMAN | LMNA | 73.8 | 63 |
|  | sp\|Q96Q06-2\|PLIN4_HUMAN | PLIN4 | 57.91 | 42 |
